# Impact of *Chamaecrista rotundifolia* cover cropping on soil elemental cycling in karst agroecosystems

**DOI:** 10.3389/fmicb.2026.1906173

**Published:** 2026-09-01

**Authors:** Zhengfu Yue, Shoushan Sheng, Liang Peng, Wei Xu, Jing Zhang, Dongming Wu, Beibei Liu, Qinfen Li, Rongshu Dong, Hanting Cheng, Yukun Zou

**Affiliations:** 1 Key Laboratory of Low-carbon Green Agriculture in Tropical Region of China, Ministry of Agriculture and Rural Affairs, Hainan Key Laboratory of Tropical Eco-Circular Agriculture, Environmental and Plant Protection Institute, Chinese Academy of Tropical Agricultural Sciences, Haikou, China; 2 National Field Observation and Research Station (Hainan Danzhou) for Tropical Agro-Ecosystem, Chinese Academy of Tropical Agricultural Sciences, Danzhou, China; 3 Key Laboratory of Tropical Translational Medicine of Ministry of Education, School of Public Health, Hainan Academy of Medical Sciences, Hainan Medical University, Haikou, Hainan, China; 4 Tropical Crops Genetic Resources Institute, Chinese Academy of Tropical Agricultural Sciences, Haikou, Hainan, China

**Keywords:** aluminum toxicity, elemental cycling, karst desertification, leguminous cover crops, microbial community

## Abstract

Karst desertification causes severe soil erosion, hydrological imbalance, and biodiversity loss, thereby threatening ecosystem resilience and agricultural productivity. Soil carbon (C), nitrogen (N), phosphorus (P), and sulfur (S) cycling underpin ecosystem stability, yet how agricultural management practices regulate multi-element cycling in degraded karst agroecosystems remains poorly understood. Here, we conducted a four-year field trial in a karst mango orchard in southwestern China and integrated metagenomic sequencing with comprehensive soil environmental profiling to investigate how *C. rotundifolia* cover cropping reshapes soil elemental cycling. Compared with conventional tillage (CK), *C. rotundifolia* cover cropping (Y) significantly (*p* < 0.05) altered topsoil microbial functional profiles and nutrient availability. Carbon cycling potentials, including gene associated with aerobic respiration (*cox1/3*), fermentation, and CO₂ assimilation—were enriched under *C. rotundifolia* cover cropping. Nitrogen cycling potentials were enhanced through increased representation of genes involved in denitrification (*nirK/S*, *nosZ*), nitrogen acquisition, and nitrate reduction (*nirA*, *narB*), while phosphorus cycling was promoted through enrichment of the PhoR-PhoB phosphate regulatory system. Sulfur transformation potentials were also altered, with increased representation of genes involved in sulfate reduction, oxidation, and sulfonate utilization. Cover cropping substantially improved soil fertility, increasing SOC (+35.4%), NH_4_^+^-N (+31.8%), TN (+35.1%), and AP (+105.9%), while reducing exchangeable Al^3+^ concentration by 60.9%. Microbial community restructuring was characterized by decreased Actinobacteriota and Chloroflexota and increased Bacteroidota and Proteobacteria, which exhibited central roles in elemental cycling networks. Mantel analyses identified exchangeable Al^3+^ as a dominant environmental constraint shaping microbial communities involved in C, N, P, and S cycling. Collectively, these findings demonstrate that *C. rotundifolia* cover cropping enhances karst soil resilience through a coupled microbial-geochemical pathway, in which improved soil chemical conditions and microbial functional reorganization jointly restore elemental cycling capacity.

## 1 Introduction

Rocky desertification represents one of the most severe forms of land degradation, characterized by extensive soil erosion, bedrock exposure, and substantial losses of soil fertility (Yan et al., 2023). This phenomenon is particularly widespread in karst landscapes, where shallow soils, discontinuous soil distribution, and highly permeable carbonate substrates create inherently fragile ecosystems that are highly sensitive to environmental disturbances (Jiang et al., 2014). Beyond physical soil loss, rocky desertification disrupts fundamental biogeochemical processes by reducing organic matter accumulation, weakening nutrient retention, and altering microbial-mediated elemental transformations, thereby constraining ecosystem recovery and agricultural sustainability. In China’s Southwest Karst region, this phenomenon has reached alarming levels, transforming once-productive farmland into rocky, uninhabitable terrain (Guo et al., 2013). The environmental consequences are profound-ranging from loss of agricultural productivity and biodiversity to increased surface runoff and regional ecological instability.

The vulnerability of karst ecosystems arises not only from shallow soils, extensive bedrock exposure, and severe soil erosion, but also from deteriorated soil chemical properties and impaired nutrient cycling. Owing to the fragmentation and discontinuous distribution of soil patches, together with rapid water infiltration through karst fissures, karst soils generally exhibit constrained and spatially heterogeneous organic matter accumulation, low nutrient-retention capacity, and pronounced spatial heterogeneity in soil fertility (Yang et al., 2023). These inherent constraints can substantially affect the storage, transformation, and bioavailability of carbon (C), nitrogen (N), phosphorus (P), and sulfur (S) (Hu et al., 2023). Carbon availability determines microbial energy supply and organic matter formation, whereas N and P constrain plant productivity and microbial growth (Wang et al., 2025). Sulfur, as an essential component of amino acids, proteins, enzymes, and cofactors, further links microbial metabolism with organic S mineralization, sulfur oxidation, and sulfate reduction or assimilation (Rebi et al., 2025). Importantly, these elemental cycles are not independent but are tightly coupled through microbial decomposition, nutrient acquisition, redox reactions, and plant–microbe interactions. Therefore, disruption of elemental coupling under rocky desertification may represent a fundamental barrier limiting soil fertility restoration and ecosystem resilience. The depletion of soil organic matter and disruption of nutrient balance associated with rocky desertification may therefore not only reduce the availability of individual nutrients but also weaken the coupling among multiple elemental cycles, ultimately constraining soil fertility recovery, vegetation establishment, and ecosystem functioning (Hu et al., 2023). Therefore, understanding the dynamics and interactions of soil C, N, P, and S cycling is fundamental to identifying the biogeochemical constraints on ecosystem recovery and to developing effective management strategies for degraded karst landscapes.

Over the past two decades, various engineering and ecological measures—such as terracing, afforestation, and chemical fertilization—have been employed to combat rocky desertification (Yao et al., 2023). However, these strategies often lack sustainability, require high labor and financial input, and may have limited long-term effectiveness in restoring ecosystem function. A growing body of research suggests that agricultural management practices that align with natural ecological processes may offer more resilient and cost-effective solutions (Qiu et al., 2024). One such practice is cover cropping—the intentional cultivation of non-cash crops to protect and enrich the soil between harvests (Souza et al., 2025). Globally, cover crops have been widely adopted for their ability to increase soil organic carbon (SOC), suppress weeds, enhance microbial diversity, reduce erosion, and improve soil structure (Hu et al., 2024). In degraded systems, especially those with nutrient-poor or erosion-prone soils, cover cropping can be a key strategy for rebuilding soil health (Huang et al., 2025). Yet, despite its proven benefits in conventional agricultural systems, the potential of cover cropping to rehabilitate karst ecosystems and mitigate rocky desertification remains poorly understood. This is in part due to the complex and heterogeneous nature of karst landscapes, where soil formation, water movement, and nutrient cycling follow distinct patterns compared to non-karst environments (Liu et al., 2024).

Recent research has begun to uncover how leguminous cover crops may help repair these disruptions. Legumes are particularly effective due to their capacity for biological nitrogen fixation and promotion of rhizosphere microbial diversity (Qiao et al., 2024). For example, planting *C. rotundifolia* (a widely used leguminous cover crop in southern China) has been associated with increased SOC levels and enhanced phosphorus availability (Zhong et al., 2018). Similarly, intercropping systems involving legumes such as Siratro or cowpea have been linked to the upregulation of functional genes related to nitrogen cycling (e.g., *nifH*, *amoA*) and carbon fixation (e.g., *cbbL*) (Xu et al., 2024). However, previous studies have largely focused on individual nutrient transformations or targeted functional genes (e.g., PCR or 16S rRNA-based methods) (Liu et al., 2025), providing limited understanding of how cover cropping restructures integrated C-N-P-S cycling networks. Metagenomics provides an opportunity to overcome these limitations by simultaneously resolving microbial taxonomic composition, functional gene potential, and interactions among multiple elemental pathways, and environmental responses at a systems level (Hu et al., 2022).

Although metagenomics has been successfully applied to study nutrient cycling in aquatic and saline ecosystems, its use in karst landscapes remains extremely limited. To date, only one PCR-based study has detected phosphorus-cycling genes (e.g., *phoD*) in karst soils (Dong et al., 2024), leaving a major gap in our understanding of how microbial communities regulate multi-element cycling in these fragile environments. To bridge this gap, we conducted a metagenomics-enabled study in a long-term field experiment located in a karst mango orchard in Nanpanjiang Town, Xingyi City, Guizhou Province, where *C. rotundifolia* has been continuously covered for several years. This system provides a unique opportunity to assess how legume cover cropping affects nutrient dynamics under real-world, rocky desertified conditions. We hypothesized that long-term *C. rotundifolia* cover cropping enhances karst soil resilience by coupling geochemical amelioration with microbial functional reorganization, thereby promoting integrated C, N, P, and S cycling. Accordingly, this study aimed to: (1) determine how long-term *C. rotundifolia* cover cropping alters microbial functional potentials involved in C, N, P, and S cycling; (2) identify microbial taxa and functional modules associated with elemental transformation networks; and (3) reveal the key environmental drivers linking soil geochemistry with microbial-mediated elemental cycling. This study provides a mechanistic basis for evaluating the potential of legume cover cropping to restore soil biogeochemical functions in rocky desertified karst ecosystems.

## 2 Materials and methods

### 2.1 Study site and experimental design

This study was conducted in Nanpanjiang Town, Xingyi City, Guizhou Province, China (113°12′40″E, 24°52′10″N), located at the junction of Guizhou, Guangxi, and Yunnan provinces. The region represents a typical karst landscape characterized by shallow soils, exposed carbonate bedrock, and high susceptibility to soil erosion. The region has a subtropical monsoon climate, with an annual precipitation of 1,000–1,500 mm, an average annual temperature of approximately 20.25 °C, and a frost-free period of about 300 days. The altitude ranges from 700 to 1,300 m, representing a low-latitude and high-elevation environment.

A long-term mango orchard experiment was established in 2008 on sloping land. Mango trees (*Mangifera indica* L.) were planted at a density of 670–820 trees/ha. In 2018, an orchard floor management experiment was initiated using a complete randomized block design. The experiment included two management strategies: (1) *Chamaecrista rotundifolia* cover cropping (Y) and (2) conventional clear tillage management (CK). The *C. rotundifolia* cover treatment was continuously maintained from 2018 until soil sampling in June 2022, representing approximately 4 years of continuous cover cropping. For the Y treatment, *C. rotundifolia*, a perennial nitrogen-fixing leguminous species widely used in southern China, was intercropped between mango trees. Seeds were sown annually in September–October at a rate of 5.5 kg/ha and a depth of 10 cm. The cover crop biomass was mowed once or twice annually and retained evenly on the soil surface as mulch to increase organic matter inputs and reduce soil erosion. In CK plots, conventional orchard management was applied, including periodic manual weeding and removal of plant residues to maintain bare soil conditions.

A total of 8 experimental plots (2 × 5 m) were randomly established, with four replicate plots for each treatment. In the present study, four plots representing the Y treatment and four plots representing the CK treatment were selected as independent biological replicates. All plots received identical fertilization and orchard management practices, including application of compound fertilizer at 1200 kg/ha/yr. (15% N, 15% P_2_O_5_, and 15% K_2_O), as well as consistent pruning, pest management, and irrigation practices.

In June 2022, soil samples were collected from each plot at two depths: surface soil (0–10 cm) and subsurface soil (10–20 cm). Five soil cores were randomly collected from each plot and thoroughly mixed to obtain one composite sample per soil layer. Samples were immediately transported to the laboratory under refrigerated conditions. After removing visible roots, gravel, and plant residues, soils were sieved through a 2 mm mesh. Each sample was divided into two subsamples: one was air-dried for physicochemical analyses, and the other was stored at −80 °C for molecular analyses.

### 2.2 Soil physicochemical analysis

Soil water content (SWC) was determined mainly by drying the soil at 105 °C for 12 h and then reweighing. Soil pH was measured using a pH meter in a soil/water (1:5 w/v) suspension. SOC was determined using K_2_Cr_2_O_7_ and H_2_SO_4_. Total nitrogen (TN) was determined by Kjeldahl method. Effective phosphorus (AP) was carried out by molybdenum-antimony resistance method. Soil exchangeable calcium (Ca) and magnesium (Mg) measurements were quantified by atomic absorption spectrophotometer,. Soil exchangeable aluminum (Al^3+^) was extracted with 1.0 mol/L KCl and titrated with 0.01 mol/L NaOH standard solution (Li et al., 2021).

### 2.3 Metagenomic sequencing and analysis

Each composite soil sample collected from an independent plot was processed separately for DNA extraction and metagenomic sequencing, and no sample pooling was performed prior to library construction. Total genomic DNA was extracted from soil samples using a magnetic bead-based protocol. DNA integrity was verified by 2% agarose gel electrophoresis, and concentration and purity were quantified using a Qubit 3.0 fluorometer (Thermo Fisher Scientific, USA). DNA was sheared to ~450 bp fragments using a Covaris M220 ultrasonicator, followed by library construction involving end-repair, A-tailing, and adapter ligation. Paired-end (2 × 150 bp) sequencing was performed on the Illumina NovaSeq 6,000 platform after library quality validation. All biological replicates were independently sequenced, resulting in a total of 16 metagenomic libraries.

Raw reads were quality-filtered using Trimmomatic v0.36 to remove adapter sequences, low-quality reads (Phred score <20), and short fragments (<75 bp) (Bolger et al., 2014). Host-derived contamination was removed by aligning reads to the human reference genome (hg38) using BWA-MEM v0.7.12.

Clean reads were assembled *de novo* using MEGAHIT v1.1.3 (Li et al., 2015), and contigs shorter than 500 bp were discarded. Gene prediction was performed using Prodigal v2.6.3 (Hyatt et al., 2010) in metagenomic mode, and predicted open reading frames (ORFs) shorter than 60 bp were removed using CD-HIT v4.6 with 95% sequence identity (Fu et al., 2012). The resulting non-redundant gene catalog was translated using the NCBI Genetic Code 11.

Taxonomic profiling was conducted using Kraken2 v2.0.6 with the PlusPF database (December 2022 release) (Lu et al., 2022), which includes bacterial, archaeal, viral, fungal, and protozoan genomes. Taxonomic abundances were refined using Bracken v2.5 (Lu et al., 2017), and species-level profiles were obtained for all samples.

Functional annotation of predicted protein sequences was performed using KofamScan based on the KEGG database and profile hidden Markov models (pHMMs) (Aramaki et al., 2020). Functional genes related to carbon, nitrogen, phosphorus, and sulfur cycling were identified using the R package CNPS.cycle,^1^ and their relative abundances were computed.

Clean reads were aligned to the non-redundant gene catalog using BWA-MEM v0.7.12 with a minimum alignment length of 50 bp and identity >95%. Gene abundance was quantified using Salmon v1.1.0 (Patro et al., 2017), and normalized as TPM (transcripts per kilobase million).

### 2.4 Statistical analysis

Beta diversity between species and functional genes was calculated using the R package vegan. The details were as follows: similarity between samples was calculated based on the Bray-Curtis distance, PCoA between samples was further calculated based on the distance matrix, and the analysis of differences between groups was tested by permutation multivariate analysis of variance (PERMANOVA) using the adonis function in the vegan package. One-way ANOVA (One-way ANOVA) and Least Significant Difference (LSD) method were used for *post hoc* tests between multiple groups. Correlation analyses were performed using IBM SPSS Statistics v27 software. Mantel test between environmental factors and microorganisms was calculated using vegan package. Spearman rank correlation coefficients (*p* < 0.05) were used to calculate correlations between microorganisms involved in the cycling pathways of the different elements (|r| > 0.8 and *p* < 0.05). Visual presentations were performed using the R packages Igraph and Gephi (v. 0.10.1). Finally, the results of the statistical analysis of the data were beautified using R (v. 4.1.3) and the R packages ggplot2 and ggcor.

## 3 Results

### 3.1 Long-term *Chamaecrista rotundifolia* cover cropping alleviates soil acidity and enhances nutrient availability

Long-term *C. rotundifolia* cover cropping significantly reshaped soil physicochemical properties, with pronounced effects observed primarily in the 0–10 cm layer (Supplementary Table S1). Compared with the uncovered control (CK), the cover-crop treatment increased soil pH from 5.35 to 5.96 and significantly increased SOC, TN, NH_4_^+^-N, AP, Ca, and Mg by 35.4, 35.1, 31.8, 106.0, 109.0, and 68.4%, respectively (*p* < 0.05). In contrast, Al^3+^ concentration decreased by 60.9%, from 0.46 to 0.18 mg/kg (*p* < 0.05). Soil water content, NO_3_^−^-N, and Fe concentration did not differ significantly between the two treatments in the surface layer, although Fe was numerically higher under Y treatment. In the 10–20 cm layer, cover cropping had no significant effect on pH, SOC, soil water content, TN, NH_4_^+^-N, NO_3_^−^-N, AP, Ca, Mg, or Fe, whereas Al^3+^ concentration decreased significantly from 0.63 to 0.42 mg/kg.

### 3.2 *Chamaecrista rotundifolia* cover cropping restructures microbial carbon-cycling potentials

**Figure 1:**
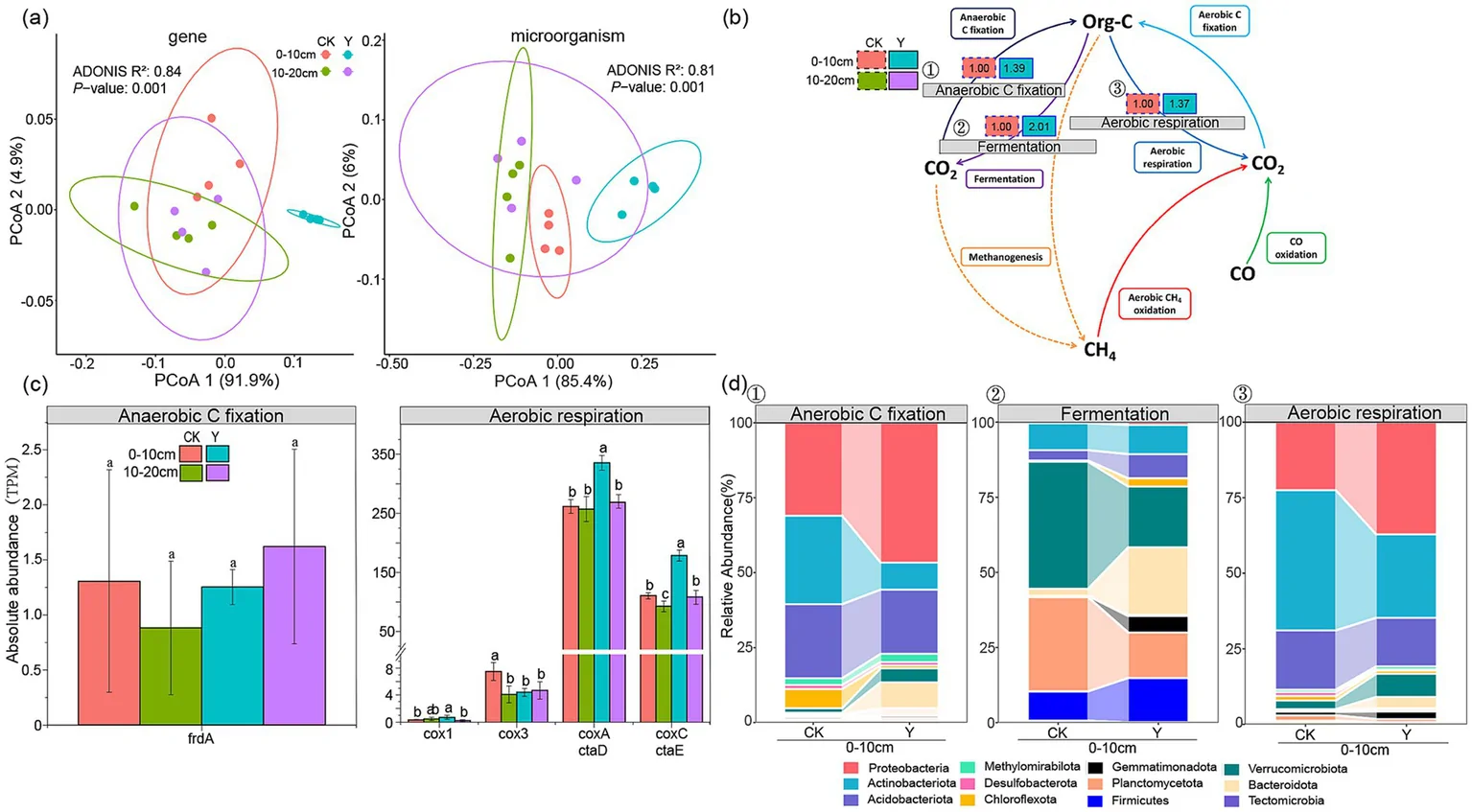
Effects of different soil cover of *C. rotundifolia* on carbon cycle functional genes and microbial communities. **(a)** PCoA principal component analysis reveals differences in carbon cycle functional genes and microbes under different treatments. Mango orchard bare ground planting treatment (CK), *C. rotundifolia* as a cover in mango orchard (Y). ADONIS indicates multivariate analysis of variance (*p* < 0.05), R^2^ indicates degree of interpretation. **(b)** Differences in the abundance of significantly performing carbon cycle processes among treatments. Values in the boxes are based on the pathway abundance of CK (0–10 cm) in the red box (1.00-fold), the dashed boxes represent the changes in CK in different soil layers, and the solid boxes represent the changes in Y in different soil layers. **(c)** Differences in functional genes exhibiting significant carbon cycling processes, TPM indicates the abundance of specific genes per million mapped reads, and letters in the boxes indicate differences between treatments (one-way ANOVA, *p* < 0.05). **(d)** Relative abundance of microbial communities exhibiting significant carbon cycling processes at the gate level. Changes in the relative abundance of microorganisms in significant pathways of carbon cycling in different soil layers.

Functional gene annotation and microbial community analyses revealed that *C. rotundifolia* cover cropping significantly altered microbial carbon-cycling functional profiles and associated communities. PCoA revealed a significant shift in both the carbon-cycling functional gene profiles (R^2^ = 0.89, *p* = 0.03) and associated microbial communities (R^2^ = 0.87, *p* = 0.027) under *C. rotundifolia* treatment (Y) compared to the uncovered control (CK) (Figures 1a,b).

Specifically, genes assigned with fermentation (+101%), anaerobic carbon assimilation (mainly frdA, +39%), and aerobic respiration (including *cox1*, *coxA*, *ctaD*, *coxC*, and *ctaE*, +37%) were enriched in surface soils under Y treatment (Figure 1c). These pathways collectively facilitate the interconversion between organic carbon and CO₂. Importantly, the enhanced abundance of these genes was positively correlated with increased SOC levels (Supplementary Table S1), suggesting functional enhancement of microbial-mediated carbon turnover under *C. rotundifolia* coverage.

Microbial community composition associated with these pathways also differed significantly between treatments (*p* < 0.05) (Figure 1d). *C. rotundifolia* covering enriched Proteobacteria, Verrucomicrobiota, and Bacteroidetes in the anaerobic and aerobic CO₂ turnover pathways, and increased the relative abundance of Acidobacteriota, Firmicutes, and Bacteroidetes in fermentation pathways. Conversely, Actinobacteriota and Planctomycetota were depleted under Y treatment, suggesting a functional reshaping of microbial consortia toward enhanced carbon metabolic activity.

Species-level correlations further revealed that *Streptacidiphilus neutrinimicus* was negatively associated with anaerobic carbon fixation (*p* < 0.05), while *RPRB01 sp003818285* and *Gp1*-*AA112 sp003225175* exhibited positive associations. Similarly, aerobic respiration was positively correlated with *Udaeobacter sp003219415* and *Sphingomicrobium sp014873795*, and negatively with *Ramlibacter sp012274225*. Fermentation activity was positively associated with *Flavisolibacter* nicotianae and *Gp1*-*AA133 sp003224995*, but negatively correlated with *JACOUH01 sp016177925* (Supplementary Figures S1a–c).

### 3.3 Cover cropping alters microbial nitrogen transformation potentials

**Figure 2:**
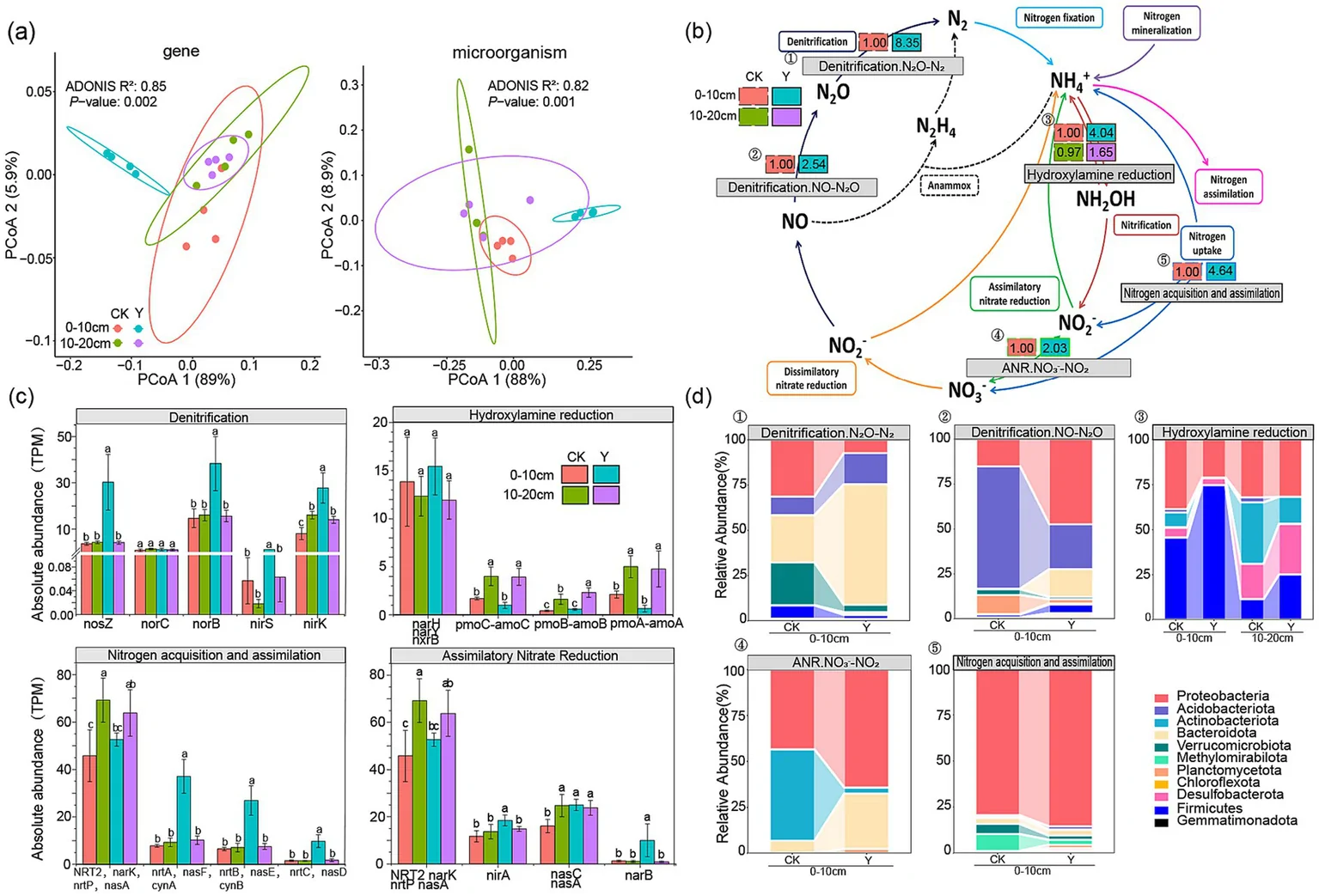
Effects of different soil cover of *C. rotundifolia* on nitrogen cycle functional genes and microbial communities. **(a)** PCoA principal component analysis reveals differences in nitrogen cycle functional genes and microbes under different treatments. Mango orchard bare ground planting treatment (CK), *C. rotundifolia* as a cover in mango orchard (Y). ADONIS indicates multivariate analysis of variance (*p* < 0.05), R^2^ indicates degree of interpretation. **(b)** Differences in the abundance of significantly performing nitrogen cycle processes among treatments. Values in the boxes are based on the pathway abundance of CK (0–10 cm) in the red box (1.00-fold), the dashed boxes represent the changes in CK in different soil layers, and the solid boxes represent the changes in Y in different soil layers. **(c)** Differences in functional genes exhibiting significant nitrogen cycling processes, TPM indicates the abundance of specific genes per million mapped reads, and letters in the boxes indicate differences between treatments (one-way ANOVA, *p* < 0.05). **(d)** Relative abundance of microbial communities exhibiting significant nitrogen cycling processes at the gate level. Changes in the relative abundance of microorganisms in significant pathways of nitrogen cycling in different soil layers.

Nitrogen-cycling functional profiles were significantly altered by *C. rotundifolia* cover cropping. PCoA revealed significant differences between the Y and CK treatments in both nitrogen cycling functional genes (R^2^ = 0.82, *p* = 0.034) and microbial communities (R^2^ = 0.89, *p* = 0.023) (Figure 2a). Specifically, the abundance of denitrification-related genes (*nosZ, norC, norB, nirS, nirK*), nitrogen acquisition and assimilation-related genes (*nrtA, nasF, cynA, nrtB, nasE, cynB, nrtC, nasD*), assimilative nitrogen reduction (*nirA, nasC, narB*), and hydroxylamine reduction associated with ammonium regeneration (NH₂OH → NH₄^+^) related genes increased 7.35, 1.54, 3.04, and 3.64-fold, respectively, under the *C. rotundifolia* cover (Figures 2b,c). These pathways are mainly involved in hydroxylamine reduction for ammonium regeneration and N_2_O reduction to N_2_, respectively. The enrichment of hydroxylamine reduction genes may contribute to enhanced ammonium regeneration potential, which was consistent with the increased NH_4_^+^ concentration observed in the soil (Supplementary Table S1). In contrast, the increased abundance of denitrification-related genes, particularly those involved in N_2_O reduction, suggests an enhanced capacity for converting reactive nitrogen intermediates into inert N_2_, potentially affecting nitrogen retention and gaseous nitrogen loss under *C. rotundifolia* cover.

Microbial community composition associated with nitrogen cycling pathways also differed significantly between treatments (Figure 2d). *C. rotundifolia* cover increased the relative abundance of Bacteroidetes involved in denitrification and assimilatory nitrate reduction pathways, and enriched Firmicutes associated with hydroxylamine reduction and ammonium regeneration. In contrast, Actinobacteriota and Verrucomicrobiota were decreased in several nitrogen transformation pathways under cover cropping. Correlation analysis revealed that NO reduction to N_2_O was positively associated with *Silvibacterium bohemicum* (*p* < 0.05), whereas N_2_O reduction to N_2_ was negatively correlated with *Sediminibacterium* sp000515315. Hydroxylamine reduction associated with ammonium regeneration (NH_2_OH → NH_4_^+^) showed a positive correlation with *Clostridium*, while nitrogen acquisition and assimilation pathways were negatively associated with *Sediminibacterium magnilacihabitans*. Additionally, assimilatory nitrate reduction (NO_3_^−^ → NH_4_^+^) was negatively correlated with *Streptomyces puniciscabiei* (Supplementary Figures S2a–e).

### 3.4 Cover cropping regulates microbial phosphorus acquisition systems

**Figure 3:**
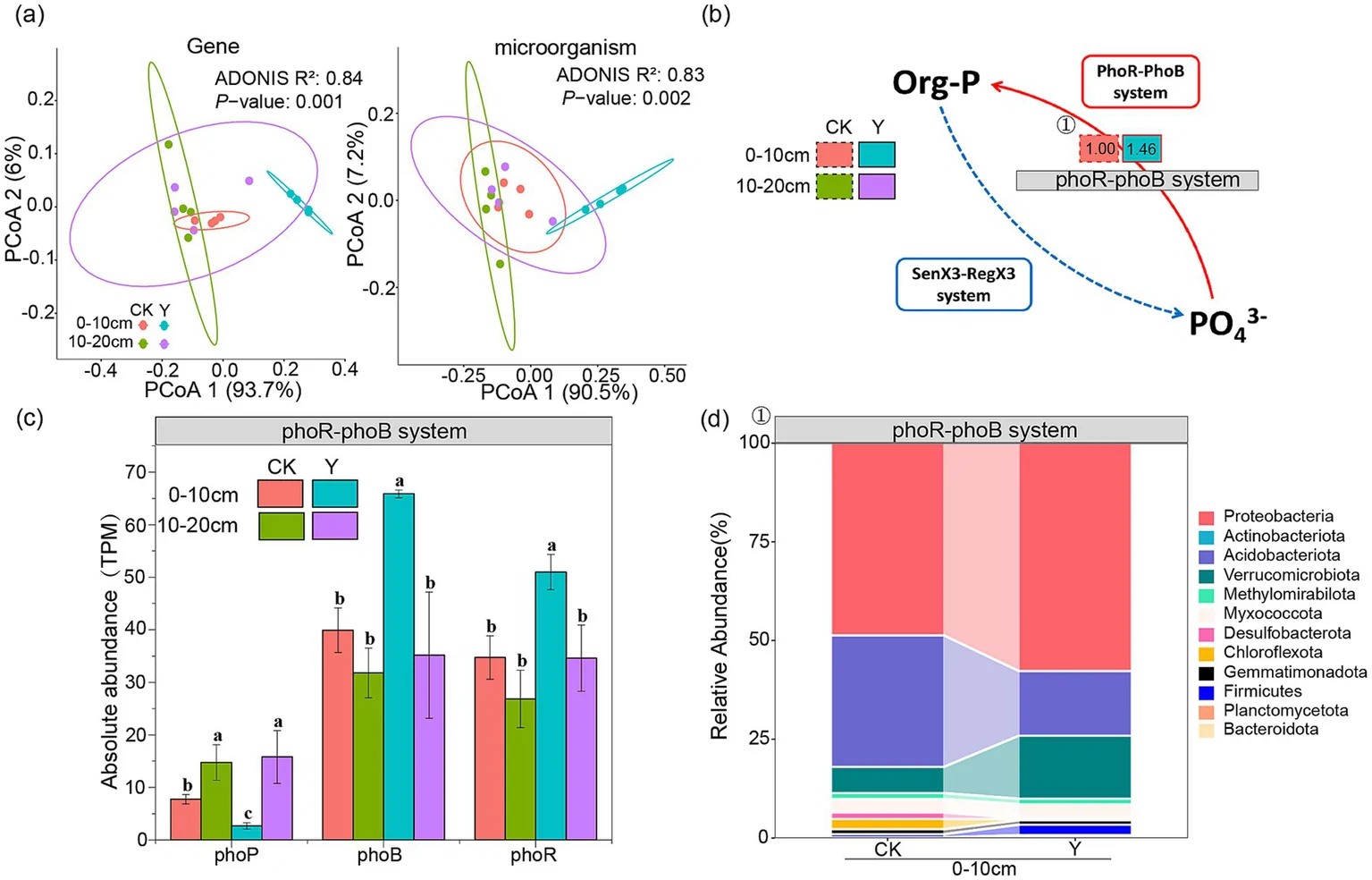
Effects of different soil cover of *C. rotundifolia* on phosphorus cycle functional genes and microbial communities. **(a)** PCoA principal component analysis reveals differences in phosphorus cycle functional genes and microbes under different treatments. Mango orchard bare ground planting treatment (CK), *C. rotundifolia* as a cover in mango orchard (Y). ADONIS indicates multivariate analysis of variance (*p* < 0.05), R^2^ indicates degree of interpretation. **(b)** Differences in the abundance of significantly performing phosphorus cycle processes among treatments. Values in the boxes are based on the pathway abundance of CK (0–10 cm) in the red box (1.00-fold), the dashed boxes represent the changes in CK in different soil layers, and the solid boxes represent the changes in Y in different soil layers. **(c)** Differences in functional genes exhibiting significant phosphorus cycling processes, TPM indicates the abundance of specific genes per million mapped reads, and letters in the boxes indicate differences between treatments (one-way ANOVA, *p* < 0.05). **(d)** Relative abundance of microbial communities exhibiting significant phosphorus cycling processes at the gate level. Changes in the relative abundance of microorganisms in significant pathways of phosphorus cycling in different soil layers.

*Chamaecrista rotundifolia* cover also modulated phosphorus (P) cycling processes in the mango orchard rhizosphere. PCoA revealed significant shifts in both P-cycling functional genes (R^2^ = 0.95, *p* = 0.032) and their associated microbial communities (R^2^ = 0.90, *p* = 0.032) (Figure 3a). Functional annotation showed that the abundance of genes within the *phoR*–*phoB* two-component regulatory system—responsible for phosphate sensing and assimilation (*phoR*, *phoB*, and *phoP*)—was increased by 46% in the surface soil of the cover treatment (Figures 3b,c). These gene clusters facilitate the conversion of inorganic phosphate into organic phosphorus pools, a trend mirrored by the observed enrichment in soil available phosphorus (Supplementary Table S1).

At the microbial level, the relative abundances of Proteobacteria and Verrucomicrobiota were significantly elevated, while Acidobacteriota declined (Figure 3d). Correlation analysis linked the abundance of phoR-phoB genes positively to *RPRB01 sp003818285* and negatively to *Acidoferrum sp003223215* (*p* < 0.05), highlighting potential microbial indicators of phosphorus bioavailability under groundcover conditions (Supplementary Figure S3).

### 3.5 Cover cropping reshapes microbial sulfur transformation potentials

**Figure 4:**
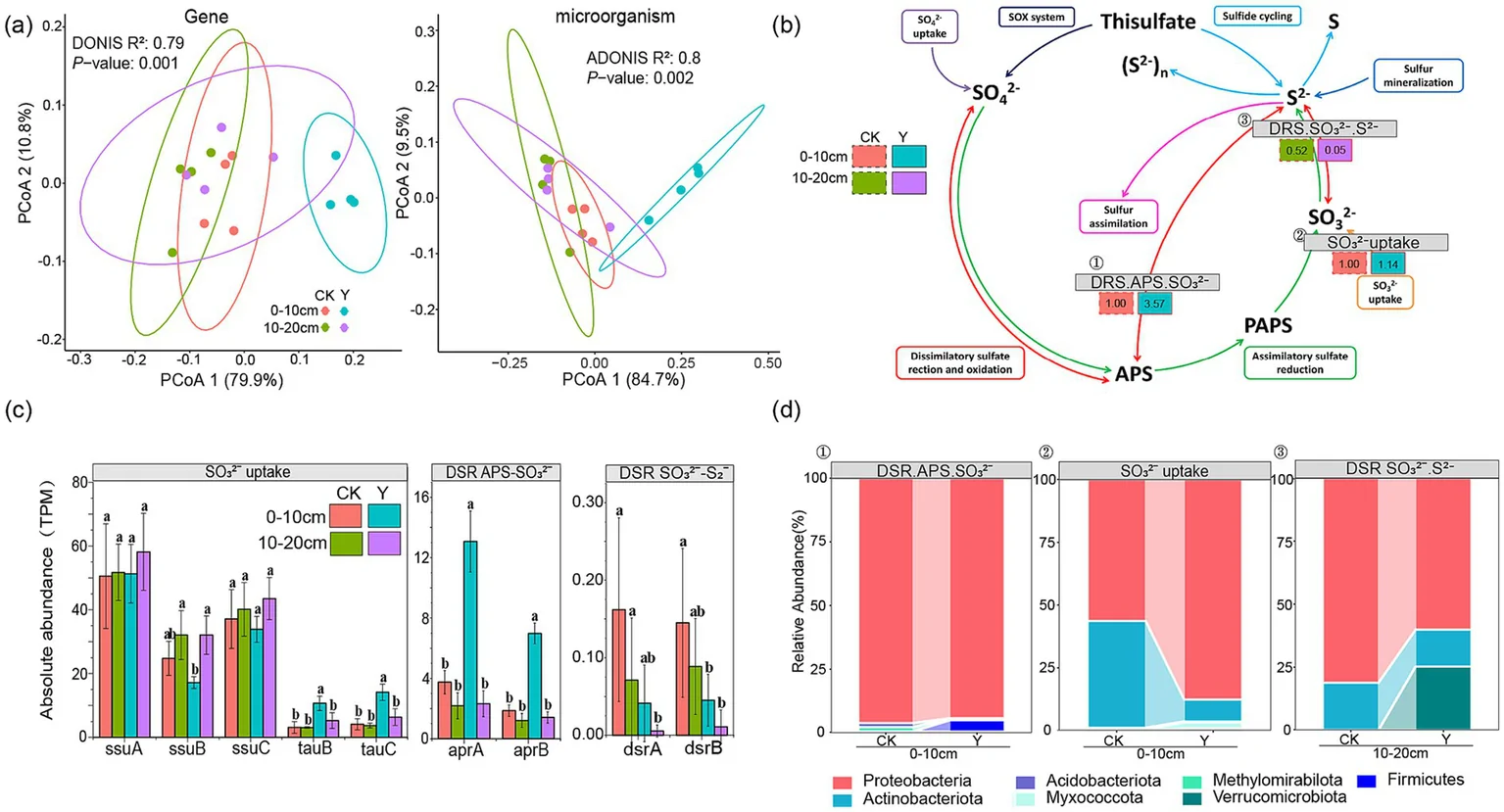
Effects of different soil cover of *C. rotundifolia* on sulfur cycle functional genes and microbial communities. **(a)** PCoA principal component analysis reveals differences in sulfur cycle functional genes and microbes under different treatments. Mango orchard bare ground planting treatment (CK), *C. rotundifolia* as a cover in mango orchard (Y). ADONIS indicates multivariate analysis of variance (*p* < 0.05), R^2^ indicates degree of interpretation. **(b)** Differences in the abundance of significantly performing sulfur cycle processes among treatments. Values in the boxes are based on the pathway abundance of CK (0–10 cm) in the red box (1.00-fold), the dashed boxes represent the changes in CK in different soil layers, and the solid boxes represent the changes in Y in different soil layers. **(c)** Differences in functional genes exhibiting significant sulfur cycling processes, TPM indicates the abundance of specific genes per million mapped reads, and letters in the boxes indicate differences between treatments (one-way ANOVA, *p* < 0.05). **(d)** Relative abundance of microbial communities exhibiting significant sulfur cycling processes at the gate level. Changes in the relative abundance of microorganisms in significant pathways of sulfur cycling in different soil layers.

Sulfur cycling was likewise significantly altered by groundcover application. Functional gene profiles differed markedly between treatments (R^2^ = 0.87, *p* = 0.026), as did the composition of sulfur-cycling microbiota (R^2^ = 0.84, *p* = 0.033) (Figure 4a). Notably, the cover treatment enhanced the abundance of assimilatory sulfate reduction (APS → SO_3_^2−^, primarily *aprA* and *aprB*) and sulfonate transport systems (*ssuA/B/C*, *tauB/C*) by 14 and 157%, respectively, in the topsoil (Figures 4b,c). In contrast, genes encoding dissimilatory sulfite reduction (SO_3_^2−^ → S^2−^via *dsrA*, *dsrB*) decreased by 47% in the subsoil layer, suggesting stratified sulfur transformation dynamics.

Microbial shifts paralleled these changes. Proteobacteria remained dominant, but Firmicutes (APS reducers), Verrucomicrobiota (SO_3_^2−^ reducers), and Myxococcota (sulfonate metabolizers) increased in the surface soil under cover treatment. Actinobacteriota declined in both dissimilatory and assimilatory pathways (Figure 4d). Correlation analysis indicated a significant positive association between aprA-driven pathways and *WYBJ01 sp009377585*, and a negative correlation with *SHVP01 sp016869765*. *Usitatibacter sp012274175* was significantly negatively correlated with SO_3_^2−^ reducers (*p* < 0.05) (Supplementary Figures S4a–c).

### 3.6 Elemental-cycling networks reveal strengthened C-N connectivity under cover cropping

**Figure 5:**
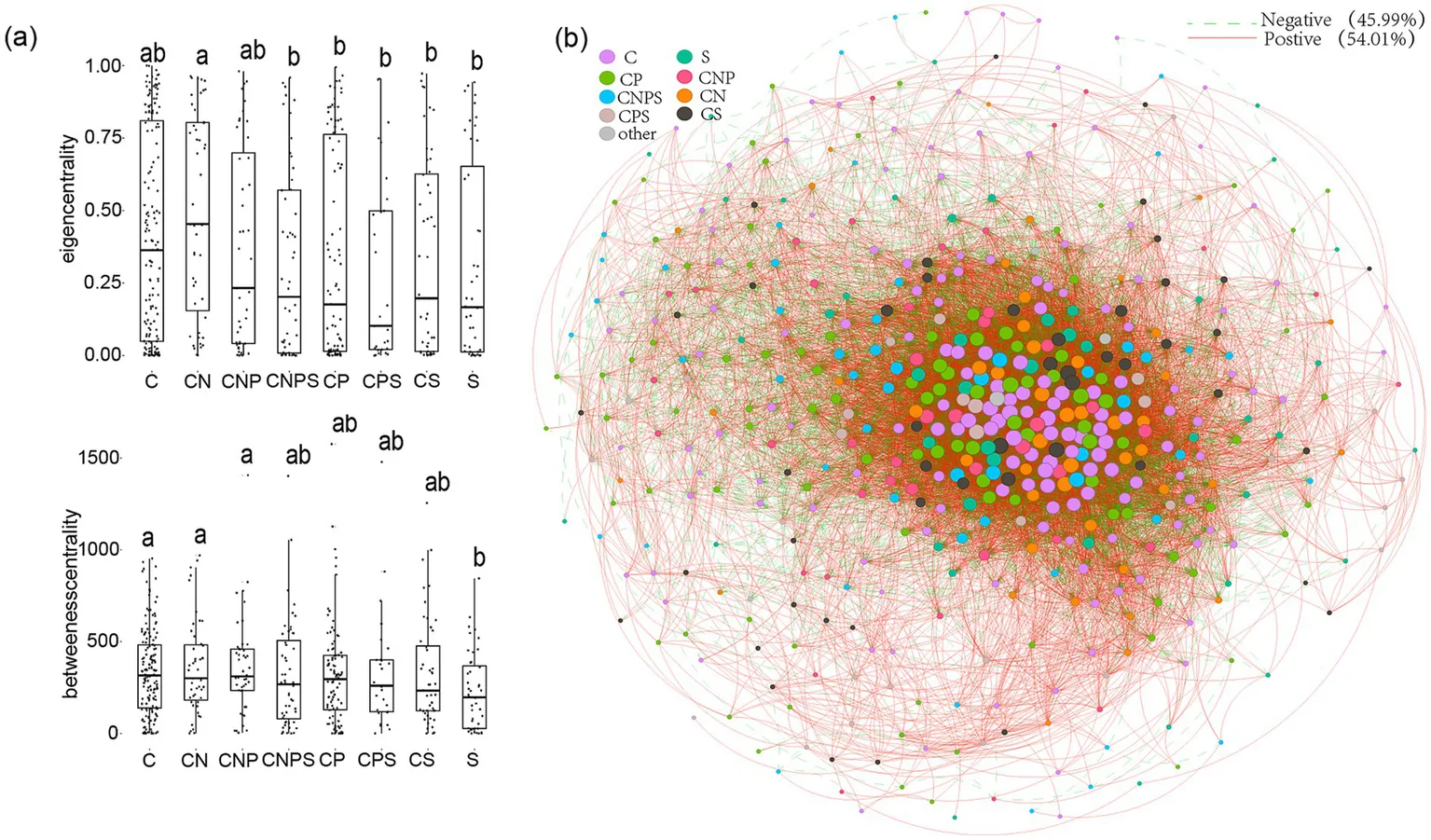
Analysis of collinear network of microorganisms associated with the geochemical cycle. **(a)** Co-occurrence network topology coefficients of microbial communities associated with soil element cycling. Betweenness centrality: Represents the number of times a node occurs in all the shortest paths, and is used to measure the node’s ability to control and transfer information in the network. Eigenvector centrality: Represents the sum of connectivity between a node and its neighboring nodes. It is used to measure the influence and importance of the node in the network. **(b)** Co-occurrence network of microbial communities associated with soil element cycling. C, N, P, and S stand for the abbreviations of carbon, nitrogen, phosphorus, and sulfur, respectively, with the red solid line indicating a positive correlation and the green dashed line indicating a negative correlation.

Co-occurrence network analysis identified 482 genera significantly associated with elemental cycling among 3,852 detected taxa, with Proteobacteria (37.55%), Acidobacteriota (22.03%), and Actinobacteriota (21.26%) dominating (Figure 5b). The global network contained 25,379 edges, of which 54.0% were positive correlations, suggesting prevalent synergistic microbial interactions.

Functional modularity analysis revealed the C cycling module as the most extensive, followed by a coupled C-P (CP) module (Figure 5). Centrality metrics—including closeness and eigenvector centrality—were significantly higher for the C and N modules than others (*p* < 0.05), indicating that microbes driving these two nutrient cycles act as ecological hubs within the network structure (Figure 5a).

### 3.7 Exchangeable Al^3+^ is a key environmental driver of elemental-cycling microbiomes

**Figure 6:**
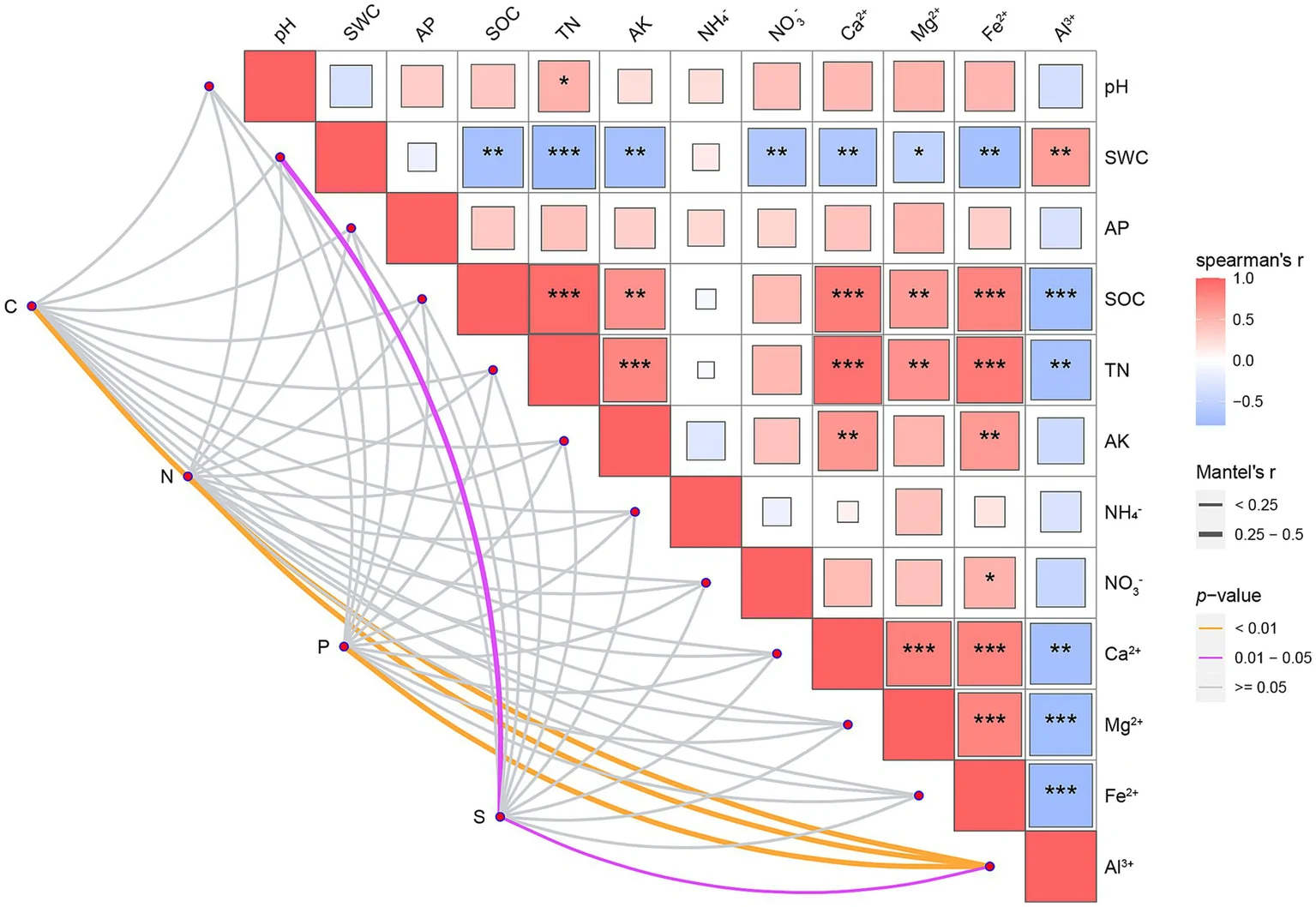
Mantel test on the relationship between geochemical cycling-related microorganisms and soil physicochemical properties. Mantel correlation physicochemical indicators with the microbial community at the genus level: The red color in the correlation graph represents positive correlation, blue color represents negative correlation, represents *p* < 0.05, represents *p* < 0.01, represents *p* < 0.001. Mantel’s R absolute value line thickness indicates the correlation size range, pink and gray indicate *p* < 0.01, *p* = 0.0 ~ 0.05, *p* ≥ 0.05, respectively.

Mantel tests revealed that SWC and Al^3+^ were significant environmental determinants of microbial communities involved in sulfur cycling (*p* < 0.05), while Al^3+^ exerted even stronger effects on microbial assemblages responsible for C, N, and P cycling (*p* < 0.01) (Figure 6). Spearman correlation analysis further illustrated a strong inverse relationship between Al^3+^ and SOC, TN, Ca, Mg, and Fe, while these nutrient pools were positively correlated with SWC (*p* < 0.05) (Figure 6).

Moreover, SOC and TN exhibited significant positive correlations with Fe, Mg, Ca, and AK, whereas NO_3_^−^ was positively associated with Fe but negatively correlated with SWC. These findings emphasize that soil geochemistry, particularly aluminum stress and moisture availability, plays a pivotal role in shaping the structure and function of microbial consortia that underpin nutrient cycling under *C*. *rotundifoli* cover.

## 4 Discussion

### 4.1 Cover cropping restores elemental cycling through enhanced resource inputs and microbial functional reorganization

The influence of *C. rotundifolia* cover cropping on elemental cycling was primarily detected in the 0–10 cm soil layer, where root activity, rhizodeposition, fine-root turnover, litter deposition, and retained mowing residues generated the strongest biological inputs (Castellano-Hinojosa et al., 2023). As a perennial leguminous cover crop, *C. rotundifolia* provided continuous inputs of root-derived labile carbon and N-rich residues, thereby increasing substrate availability for microbial growth and nutrient transformation (Canarini et al., 2019). These concentrated surface inputs provided the primary biological basis for the coordinated responses of C, N, P, and S cycling.

For carbon cycling, enrichment of fermentation- and aerobic-respiration-related genes, including *cox1*, *coxA*, *ctaD*, *coxC*, and *ctaE*, indicated an increased microbial capacity for organic carbon transformation under cover cropping. This response is consistent with enhanced microbial turnover of root-derived compounds and plant residues rather than suppression of respiratory activity (Kallenbach et al., 2016; Sokol et al., 2019). The observed increase in SOC likely resulted from a balance between enhanced plant-derived carbon inputs, microbial transformation into persistent organic matter, and reduced erosion-mediated carbon loss (Tang et al., 2021). Proteobacteria were strongly associated with both aerobic respiration and anaerobic carbon-transformation pathways, suggesting that metabolically flexible members of this phylum responded to increased rhizosphere resource availability (Garrido-Sanz and Keel, 2025). Enrichment of genes assigned to anaerobic carbon-fixation-related pathways further indicated altered carbon-assimilation potential in oxygen-limited microsites. However, because several constituent genes also participate in other anaerobic processes, this enrichment cannot be equated directly with greater net CO_2_ fixation.

Nitrogen cycling appeared to respond to the coupled input of residue-derived C and N. Increased NH_4_^+^-N concentrations, together with greater representation of hydroxylamine-associated genes, suggested shifts in microbial nitrogen transformation potential. Broad annotations of hydroxylamine-related genes do not resolve reaction direction and should therefore be interpreted only as evidence of a shift in pathway representation (Caranto and Lancaster, 2017). More clearly, the increase in *nosZ* and decline in the (*nirS* + *nirK*)/*nosZ* ratio from 2.25 to 0.96 indicated that genes involved in N_2_O reduction became more strongly represented relative to nitrite-reduction genes within the denitrification gene pool. Labile carbon supplied by roots and decomposing residues may have supported heterotrophic denitrification, while the higher pH may have alleviated acid constraints on N_2_O reduction (Deveautour et al., 2022). This pattern suggests an increased representation of genes potentially associated with complete denitrification, although it does not demonstrate reduced N_2_O emissions. The associations of Firmicutes with fermentative functions and Bacteroidota with denitrification further connected residue decomposition with downstream N transformations.

Phosphorus responses reflected the combined effects of microbial phosphate acquisition strategies and improved soil chemical conditions under cover cropping. The enrichment of *phoR* and *phoB* indicated increased microbial potential for phosphate sensing, regulation, and acquisition (Santos-Beneit, 2015). Root-derived compounds and decomposing residues may also have supported microbial growth and favored taxa capable of acquiring P from organic or sparingly available pools (Tang et al., 2021). The increase in surface-soil TP should not be interpreted as evidence of P mineralization, because mineralization changes P forms rather than the total P stock. Instead, higher TP probably reflected upward biological redistribution of P through plant uptake and residue return, together with reduced erosion-mediated loss of P-rich surface soil (Tang et al., 2021). Changes in P availability were additionally linked to acidity amelioration and Al dynamics, which are considered collectively in Section 4.3.

For sulfur cycling, changes in *aprA/aprB* and *dsrA/dsrB* indicated redistribution of genetic potential among APS and sulfite transformation steps. AprAB- and DsrAB-related proteins can participate in either reductive or oxidative sulfur pathways depending on their phylogenetic and genomic contexts (Mateos et al., 2023). The observed changes therefore do not establish pathway direction, but they are consistent with altered organic-substrate supply, microbial S demand, and redox conditions in surface-soil microsites. Therefore, *C. rotundifolia* altered sulfur cycling primarily by modifying the ecological context of sulfur-transforming microorganisms rather than uniformly stimulating or suppressing specific pathways.

### 4.2 Cover cropping strengthens microbial connectivity among coupled elemental cycles

Carbon-cycling microorganisms formed a central component of the integrated elemental network because carbon availability regulates microbial energy supply, biomass formation, and downstream nutrient transformations., and serves as an electron donor for many reductive N- and S-transforming processes (Sokol et al., 2022). The C-N network showed higher connectivity and lower modularity than the C-P and C-S networks, indicating dense and weakly compartmentalized associations between C- and N-cycling taxa. This structure is consistent with the close biochemical linkage between decomposition of relatively N-rich legume residues, mineral-N release, microbial growth, and heterotrophic N transformations (White et al., 2022).

Proteobacteria, Actinobacteriota, and Acidobacteriota dominated C-N associations but probably contributed through different ecological strategies. Many Proteobacteria respond rapidly to labile rhizosphere substrates, some Actinobacteriota degrade structurally complex plant compounds, and Acidobacteriota contain lineages adapted to contrasting pH and resource conditions (Ling et al., 2022; Shinde et al., 2022; Sikorski et al., 2022). Their joint occurrence suggests that C-N coupling involved both rapid utilization of soluble compounds and subsequent decomposition of more complex residues. C-P coupling was mainly associated with Acidobacteriota and Proteobacteria and probably integrated microbial P acquisition with mineral-surface processes. Carbon inputs can simultaneously increase microbial P demand, stimulate organic-P turnover, and alter the activity of P-acquiring microorganisms (Huang et al., 2024). The resulting network therefore likely reflected both shared responses to plant-derived substrates and interactions between biological P demand and soil P accessibility (Lei et al., 2024). In contrast, C-S associations were more strongly compartmentalized. Their biochemical basis may lie in the coupling of heterotrophic sulfate reduction to organic-carbon oxidation and of sulfur oxidation to chemolithotrophic carbon assimilation (Dyksma and Pester, 2023). The higher modularity of the C-S network indicates more clearly separated association clusters than in the C-N network, but does not by itself prove greater functional specialization.

Across the integrated C-N-P-S network, Proteobacteria occupied central positions, probably because this diverse phylum contains taxa involved in multiple respiratory and nutrient-transformation pathways. Actinobacteriota acted as secondary hubs and may have connected plant-residue decomposition with nutrient release (Ling et al., 2022; Shinde et al., 2022). These central positions identify candidate taxa that may integrate multiple elemental cycles, but they do not demonstrate direct metabolic exchange. Network links may also result from common environmental preferences, shared responses to substrate inputs, or indirect associations.

### 4.3 Soil acidity and Al toxicity represent key geochemical constraints regulating microbial restoration

Mantel analyses identified soil acidity-related variables, particularly pH and exchangeable Al^3+^, as major environmental factors associated with microbial communities involved in elemental cycling. These variables should be regarded as components of a common environmental control axis rather than independent drivers. Soil acidification increases Al solubility and exchangeability, whereas increasing pH shifts Al toward hydrolyzed, organically complexed, or otherwise less labile forms (Zhang et al., 2022). In this study, *C. rotundifolia* cover increased soil pH from 5.35 to 5.96 and reduced exchangeable Al^3+^ from 0.46 to 0.18 mg/kg, indicating substantial amelioration of the acidic surface soil. Several interconnected mechanisms may explain this geochemical shift. Plant-residue return can supply alkalinity associated with organic salts and recycle base cations such as Ca^2+^, Mg^2+^, and K^+^ to the soil surface. SOC accumulation may further increase cation-exchange and pH-buffering capacities, while organic functional groups can bind protons and complex Al (Li et al., 2022; Zhang et al., 2023). These effects may collectively offset proton inputs associated with rhizosphere nutrient uptake and nitrification of ammonium derived from residue mineralization or biologically fixed N. The observed increase in pH was therefore more likely produced by the combined effects of residue-derived alkalinity, base-cation recycling, organic-matter buffering, and reduced Al mobilization than by a single mechanism.

The decline in exchangeable Al^3+^ may have affected elemental cycling through both biological and geochemical pathways. Biologically, lower Al stress may have relaxed environmental filtering, allowing more microbial taxa to persist and remain active (Zhang et al., 2022). This interpretation is consistent with the increase in Shannon diversity and the strong association between exchangeable Al^3+^ and microbial community variation. Reduced Al toxicity may also favor root development and microbial nutrient acquisition. Geochemically, acidity amelioration and changes in exchangeable and other reactive Al fractions may decrease Al-associated phosphate sorption or precipitation, thereby improving P accessibility (Lei et al., 2024). This mechanism provides a physicochemical complement to microbial P acquisition and helps explain why the P response cannot be attributed solely to changes in P-cycling genes. Improved root growth could subsequently increase rhizodeposition and residue inputs, potentially reinforcing microbial growth and nutrient turnover (Philippot et al., 2024). However, because root growth and rhizodeposition were not measured, this plant–soil-microbe feedback remains a mechanistic hypothesis. Overall, *C. rotundifolia* cover cropping enhanced karst soil functional resilience through two interconnected pathways: increasing plant-derived resource inputs that supported microbial nutrient transformation, and alleviating acidity-driven Al constraints that shaped microbial community assembly and nutrient accessibility. The overlap of these biological and physicochemical effects in surface soil explains the pronounced depth dependence of the observed responses.

### 4.4 Ecological implications, limitations, and future directions

The coordinated responses of C, N, P, and S cycling observed in the surface soil have several implications for orchard management in karst regions. First, *C. rotundifolia* cover can provide continuous plant-derived carbon inputs and return relatively N-rich residues to the soil, thereby supporting microbial nutrient turnover and SOC accumulation. Second, the increase in soil pH and decline in exchangeable Al^3+^ indicate that this cover crop may help alleviate two major constraints of acidic karst soils: Al toxicity and limited P accessibility. Third, because the effects were concentrated in the 0–10 cm soil layer, retaining mown biomass and minimizing disturbance of the soil surface may be important for maintaining these benefits. In sloping orchards, vegetation cover may also reduce runoff and the erosion-mediated loss of organic matter and nutrients. These functions suggest that *C. rotundifolia* could serve as a biological complement to liming and fertilizer management, rather than merely as a ground-cover species. Its integration into orchard management may simultaneously contribute to soil conservation, organic matter accumulation, acidity amelioration, and nutrient recycling, potentially reducing long-term dependence on external soil amendments.

However, several limitations define the evidence boundaries of this study. Metagenomic DNA reflects functional potential rather than gene expression, process rates, or net elemental fluxes (Rocca et al., 2015), and broad functional annotations cannot unambiguously resolve the direction of hydroxylamine metabolism, fumarate-related carbon metabolism, or AprAB/DsrAB-mediated sulfur transformations. Co-occurrence networks and Mantel tests identify statistical associations but cannot establish direct metabolic exchange or causality (Gao et al., 2022). Therefore, the agronomic and environmental benefits inferred here require validation through long-term field measurements integrating crop productivity, nutrient-use efficiency, erosion control, greenhouse-gas emissions, and economic performance. Future studies should further combine process-rate measurements and stable-isotope tracing with metatranscriptomics, MAG-resolved metabolic reconstruction, and targeted soil-geochemical analyses. Such evidence will be necessary to determine the conditions under which *C. rotundifolia* cover can be effectively incorporated into sustainable management strategies for acidic karst orchards.

## 5 Conclusion

Long-term *C. rotundifolia* cover cropping reshaped microbial-mediated elemental cycling in karst mango orchard soils, with effects primarily concentrated in surface soil. Rather than acting solely as a nutrient input strategy, leguminous cover cropping promoted soil functional recovery through coordinated biological and geochemical mechanisms. *C. rotundifolia* covering significantly altered elemental cycling processes in karst mango orchard topsoils, primarily enhancing anaerobic carbon assimilation (C cycle), denitrification-related processes (N cycle, 7.35-fold increase in gene abundance), the phoR-phoB phosphate regulatory system (P cycle), and sulfur transformation pathways, including APS reduction-related processes and sulfonate transport (S cycle), while suppressing dissimilatory sulfite reduction. Furthermore, Proteobacteria and Bacteroidetes were identified as important microbial groups associated with coupled C–N–P–S cycling, and their enrichment was significantly correlated with changes in soil nutrient availability (*p* < 0.05). *C. rotundifolia* cover modulates soil nutrient dynamics through dual chemical-biological mechanisms: (1) pH elevation (5.35 → 5.96) and Al^3+^ activity reduction (0.46 → 0.18 mg/kg) enhance nutrient desorption; (2) Enriched geochemical cycling taxa (e.g., Proteobacteria, Bacteroidetes) accelerate nutrient biotransformation. Our findings reveal that leguminous cover crops can restore degraded karst agroecosystems by coupling soil chemical amelioration with microbial functional reorganization, providing a mechanistic basis for sustainable soil management in fragile carbonate landscapes.

## Data Availability

The metagenomic sequencing data generated in this study have been deposited in the NCBI Sequence Read Archive (SRA) under BioProject accession number PRJNA1513441.

## Footnotes

1
https://github.com/yuezhengfu/CNPS.cycle
