# Supplementary material for: Impact of Chamaecrista rotundifolia cover cropping on soil elemental cycling in karst agroecosystems
Source: Front Microbiol. 2026 Sep 1;17:1906173. doi: 10.3389/fmicb.2026.1906173 (PMC13574786; doi:10.3389/fmicb.2026.1906173)
Supplement: Supplementary file 1 [file Supplementary_file_1.DOCX]

**Impact of *Chamaecrista rotundifolia* Cover Cropping on Soil Elemental Cycling in Karst Agroecosystems**

Zhengfu Yue^1,4#^, Shoushan Sheng^1,4#^, Liang Peng^1,4^, Wei Xu^1,4^, Jing Zhang^2^, Dongming Wu^1,4^, Beibei Liu^1,4^, Qinfen Li^1,4^, Rongshu Dong^3^, Hanting Cheng^1,4*^, Yukun Zou^1,4*^

^1^ Key Laboratory of Low-carbon Green Agriculture in Tropical region of China, Ministry of Agriculture and Rural Affairs; Hainan Key Laboratory of Tropical Eco-Circular Agriculture, Environmental and Plant Protection Institute, Chinese Academy of Tropical Agricultural Sciences, Haikou 571101, China

^2^ Key Laboratory of Tropical Translational Medicine of Ministry of Education, School of Public Health, Hainan Academy of Medical Sciences, Hainan Medical University, Haikou, Hainan 571199, China

^3^ Tropical Crops Genetic Resources Institute, Chinese Academy of Tropical Agricultural Sciences, Haikou, Hainan 571101, China

^4^ Hainan Danzhou Tropical Agro-ecosystem National Observation and Research Station, Chinese Academy of Tropical Agricultural Sciences, Danzhou 571737, China

\# These authors contributed equally to this work

**Corresponding author:** Hanting Cheng^1,4^ and Yukun Zou^1,4^

E-mail: [hantingcheng@catas.cn](mailto:hantingcheng@catas.cn); [yukun.zou@catas.cn](mailto:yukun.zou@catas.cn)

**Table S1** Effects of *chamaecrista rotundifoli* cover on soil physicochemical properties in different soil layers.

|  | Treatment | pH | SOC（g/kg） | SWC  % | TN  （g/kg） | NH_4_^+^-N  (mg/kg) | NO_3_^—^N  (mg/kg) | AP  (mg/kg) | Ca  （g/kg） | Mg  （g/kg） | Fe  （mg/kg） | Al  （mg/kg） |
| --- | --- | --- | --- | --- | --- | --- | --- | --- | --- | --- | --- | --- |
| 0-10cm | CK | 5.35±0.16b | 10.04±2.19b | 22.97±1.97b | 0.94±0.07cd | 10.65±2.74c | 0.67±0.02b | 2.67±1.47b | 1.44±0.09c | 0.19±0.01d | 48.80±11.58ab | 0.46±0.02bc |
|  | Y | 5.96±0.22a | 13.59±1.07a | 22.49±1.09b | 1.27±0.08ab | 14.04±3.38ab | 0.85±0.25ab | 5.50±1.56a | 3.01±0.38b | 0.32±0.05ab | 68.17±6.71a | 0.18±0.03e |
| 10-20cm | CK | 5.47±0.19b | 6.50±1.78d | 26.45±1.08a | 0.71±0.16d | 11.87±1.47bc | 0.60±0.07b | 2.40±1.09b | 1.26±0.29c | 0.16±0.02d | 35.88±4.12ab | 0.63±0.11a |
|  | Y | 5.40±0.20b | 6.94±2.02cd | 26.64±1.33a | 0.74±0.17d | 16.54±1.60ab | 0.58±0.08b | 3.25±1.65b | 1.50±0.48c | 0.19±0.02d | 40.90±8.96b | 0.42±0.08bc |

SOC (Soil organic carbon), Soil water content (SWC), Total nitrogen (TN), Ammonium nitrogen (NH_4_^+^-N), nitrate nitrogen (NO_3_^-^-N)

**Fig.S1** The Spearman correlation between the significant pathways of the carbon cycle under the coverage of c*hamaecrista rotundifoli* and microorganisms at the genus level(a-c).the red color in the correlation graph represents positive correlation, blue color represents negative correlation, * represents *p*<0.05, ** represents *p*<0.01 , *** represents *p*<0.001.

**Fig.S2** The Spearman correlation between the significant pathways of the nitrogen cycle under the coverage of c*hamaecrista rotundifoli* and microorganisms at the genus level(a-c).the red color in the correlation graph represents positive correlation, blue color represents negative correlation, * represents *p*<0.05, ** represents *p*<0.01 , *** represents *p*<0.001.

**Fig.S3** The Spearman correlation between the significant pathways of the phosphorus cycle under the coverage of c*hamaecrista rotundifoli* and microorganisms at the genus level(a-c).the red color in the correlation graph represents positive correlation, blue color represents negative correlation, * represents *p*<0.05, ** represents *p*<0.01 , *** represents *p*<0.001.

**Fig.S4** The Spearman correlation between the significant pathways of the sulfur cycle under the coverage of C*hamaecrista rotundifoli* and microorganisms at the genus level(a-c).the red color in the correlation graph represents positive correlation, blue color represents negative correlation, * represents *p*<0.05, ** represents *p*<0.01 , *** represents *p*<0.001.
